# The rationale of opportunistic bilateral salpingectomies (OBS) during benign gynaecological and obstetric surgery: a consensus text of the Flemish Society of Obstetrics and Gynaecology (VVOG)

**Published:** 2019-06

**Authors:** WAA Tjalma, JJA Bosteels, I Cooremans, S Cosyns, M De Greve, BP De Vree, D Debruyne, ET De Jonge, EJI Desmedt, P Dubois, T Faes, M Francx, T Hamerlynck, AP Makar, AS Maryns, I Michiels, G Orye, L Platteeuw, B Pouseele, V Schutyser, A Segaert, M Stevens, C Tomassetti, XB Trinh, P Tummers, SGK van Calenbergh, PA van Dam, B Van Herendael, R Vanspauwen, IB Vergote, J Verguts, K Watty, S Weyers

**Affiliations:** Antwerp University Hospital – University of Antwerp, Antwerpen;; Imelda Hospital, Bonheiden;; AZ Delta campus Menen, Menen;; Vrije Universiteit Brussel - Universitair Ziekenhuis Brussel, Brussel;; AZ Turnhout, Turnhout;; ZNA Middelheim, Antwerpen;; AZ Groeninge, Kortrijk;; ZOL, Genk - Hasselt University, Hasselt;; GZA, Wilrijk;; KLINA, Brasschaat;; AZ Sint Blasius Dendermonde, Dendermonde;; Universitair Ziekenhuis Gent, Gent;; Sint-Trudo, Sint-Truiden;; Jessa Ziekenhuis, Hasselt;; OLV van Lourdes Ziekenenhuis, Waregem;; AZ Maria Middelares, Gent;; AZ Rivierenland campus Bornem, Bornem;; UZ Leuven, Leuven;; ZNA Stuivenberg, Antwerpen;; AZ Delta campus Roeselare, Roeselare, Belgium.

**Keywords:** salpingectomy, ovarian cancer, risk-reducing, reduction, prevention, menopause, opportunistic, prophylactic, benefits, risk, safety

## Abstract

Ovarian cancer (OC), is a disease difficult to diagnose in an early stage implicating a poor prognosis. The 5-year overall survival in Belgium has not changed in the last 18 years and remains 44 %. There is no effective screening method (secondary prevention) to detect ovarian cancer at an early stage. Primary prevention of ovarian cancer came in the picture through the paradigm shift that the fallopian tube is often the origin of ovarian cancer and not the ovary itself. Opportunistic bilateral salpingectomy (OBS) during benign gynaecological and obstetric surgery might have the potential to reduce the risk of ovarian cancer by as much as 65 %. Bilateral risk-reducing salpingectomy during a benign procedure is feasible, safe, appears to have no impact on the ovarian function and seems to be cost effective. The key question is whether we should wait for a RCT or implement OBS directly in our daily practice. Guidelines regarding OBS within our societies are therefore urgently needed. Our recommendation is to inform all women without a child wish, undergoing a benign gynaecological or obstetrical surgical procedure about the pro’s and the con’s of OBS and advise a bilateral salpingectomy. Furthermore, there is an urgent need for a prospective registry of OBS. The present article is the consensus text of the Flemish Society of Obstetrics and Gynaecology (VVOG) regarding OBS.

## Introduction

### 

The life time risk of developing ovarian cancer (OC) is 1.4%. In 2016, 752 Belgian women were diagnosed with OC at a mean age of 65.6 years (Belgian Cancer Registry 2016, 2016). OC is the 8 th (2%) most prevalent cancer and the 5 th (6%) most common cause of cancer-related death among Belgian women (Belgian Cancer Registry 2015, 2015). The past 2 decades the 5-year survival hasn’t changed and is around 44 % (Belgian Cancer Registry 2015, 2015; Belgian Cancer Registry 2016, 2016). In the same period the Belgian Cancer Registry indicated a 2.9 decline in incidence (the age-standardised incidence rates in 2004 was 9.7 and in 2016 it became 6.8). For 2025 it is expected that age-standardized rate will be 5.6 [CI 95 % 5.1;6.1], this translates into 716 [95 % CI 658;774] females who will receive the diagnosis of OC (Belgian Cancer Registry 2016, 2016). The decline can be partially contributed to the use of oral contraceptives and hormonal IUDs.

The majority of the patients present in an advanced stage and the stage distribution in the period 2004 – 2016 has not changed (Table I) (Belgian Cancer Registry 2016, 2016). Based on recent molecular and genetic data it became apparent that epithelial OC is a heterogenic disease. Epithelial OC has therefore been divided into 2 types. Type 1 includes mucinous, endometrioid and clear cell carcinomas, while type 2 includes serous carcinomas and adenocarcinoma NOS (not otherwise specified). Tumour subtypes have an important influence on stage presentation at diagnosis and survival. Type 2 are mainly (>75%) diagnosed in an advanced stage, while type 1 are mainly (>65%) diagnosed in an early stage (Table I) (Belgian Cancer Registry 2015, 2015). The histological subtype of OC division is very unequal with one fifth being type 1 and four fifths being type 2 (Table I). The subtype incidence has changed slowly the last 14 years and at present for serous carcinoma it is 4 per 100.000 while for each of the other cancers it is below 1 per 100.000.

**Table I:** — OC in general, OC subtypes and stage distribution.

		Stage division			
		I	II	III	IV
OC in general	100%	24%	7%	37%	32%
OC Subtypes					
Type 1 OC	22%				
- Mucinous	9 %	71 %	4 %	14 %	12 %
- Endometroid	8 %	61 %	11 %	18 %	11 %
- Clear cell	5 %	54 %	9 %	24 %	14 %
Type 2 OC	78 %				
Serous carcinomas	68 %	13 %	9 %	47 %	31 %
Adenocarcinoma NOS	10 %	16 %	3 %	39 %	44 %

The primary reason for the high mortality in OC is the detection of the disease in an advanced stage. When OC is detected in stage I the patient can be cured in 90 % of the cases. Unfortunately, today there is no adequate screening (secondary prevention) for OC in asymptomatic women with an average risk on OC. The results of the randomised controlled trials and meta-analyses are clear: different screening modalities (transvaginal ultrasound, cancer antigen 125 (CA-125) testing, or their combination) did not lead to a reduction in mortality (Reade et al., 2013). On the contrary screening asymptomatic women will lead to an increased morbidity due to screen-induced explorative surgery and related psychologic distress (Elias et al., 2018; Henderson et al., 2018; Reade et al., 2013). In women with a proven high genetic risk of developing ovarian, peritoneal or tubal cancer it is considered established practice to offer a prophylactic risk-reducing bilateral salpingo-oophorectomy (PBSO) after completion of the child wish (primary prevention). Removing the ovaries and tubes is very effective in reducing ovarian, peritoneal and tubal cancer, but could have a morbidity related to premature onset of menopause. The morbidity can be reduced by hormonal replacement therapy until the age of natural menopause. The morbidity depends of course when the ovaries are removed. For BRCA2, RAD51C and BRIP1 the surgery can be performed relatively close to menopause. A PBSO on the otherhand for BRCA1 should be advised at a young age.

Histologic findings from these PBSO patients revealed that some women had precancerous lesions in the fimbriae (tubal intraepithelial carcinoma or TIC) (Crum et al., 2007b, 2007a; Kim et al., 2018). The latter appeared particularly true for serous tubal intraepithelial carcinoma (STIC), which is assumed to be a precursor of high-grade serous cancer (HGSC). These findings caused a paradigm shift as the fallopian tube epithelium rather than the ovarian epithelial surface was regarded as the origin of epithelial high-grade serous OC (J. Kim et al., 2018). Dicer- Pten DKO (Double Knock Out) mice models showed that removing the fallopian tube could prevent the development of OC (Kim et al., 2012). Based on these clinical and laboratory findings the idea arose that primary prevention by bilateral salpingectomy could reduce the incidence of OC in women with an average risk of OC. Presentations and discussions at national and international meetings in the low countries regarding this topic indicated the necessity for a consensus guideline in daily practice (Tjalma, 2018, 2017). This article will describe the rationale for opportunistic bilateral salpingectomy (OBS) during benign gynaecological and obstetric surgery. The goal is to establish a consensus regarding OBS among members of the Flemish Society of Obstetrics and Gynaecology.

### Origin of ovarian cancer according to subtype

OC can be divided into two types (Table I) (Dhakal et al., 2017). The clinical behaviour of the types reflects their genetic set-up and immunohistochemical features (Piszczek et al., 2018). In OC pathology, unlike most cancers that become less differentiated during their neoplastic makeover, advanced epithelial OC ovarian display four distinct histological types that resemble the well-differentiated normal cells within the Mullerian system: serous type (fallopian tube origin), endometrioid type (endometrial origin), mucinous type (endocervical origin), and clear cell type (vaginal epithelial origin). OC from the Mullerian system originate from the ovary. The high-grade serous carcinomas (HGSC) originate from the STIC in the distal end of the tube (fimbriae) and not from the ovary. This is supported by data that no STIC or precursor lesions were found on the ovaries or peritoneum (Callahan et al., 2007; Cass et al., 2005; Kindelberger et al., 2007; Mittal et al., 2016; Piek et al., 2001). The shared sequence specific TP52 mutations by STICs and metastatic HGSCs and the shared molecular profiling by HGSC and the fallopian tube further support this finding (Carlson et al., 2008; Chene et al., 2013; Kim et al., 2018; Kindelberger et al., 2007; Kurman and Shih, 2011; Labidi-Galy et al., 2017; Lee et al., 2007; Mallen et al., 2018; Marquez et al., 2005; Mehrad et al., 2010; Przybycin et al., 2010). Further proof is given by the finding that biomarkers of HGSC are more inclined to relate to those of fallopian tube than to ovarian or peritoneal tissue (Erickson et al., 2013; Kurman and Shih, 2011; Mittal et al., 2016; Piszczek et al., 2018). The STICs transform into an invasive tubal cancer and by direct implantation they cause metastasis on the ovary and/or peritoneum (McCluggage et al., 2017; Mittal et al., 2016) .

It is believed that ovarian and peritoneal endometriosis are caused by the direct implantation of endometrial tissue. Whereas retrograde menstruation (blood and endometrial cells entering the peritoneal cavity through the fallopian tubes) occurs in about 90% of women, other mechanisms such as immune dysfunction and/or endometrial stem cells are probably involved in the implantation of endometrial cells and subsequent lesion formation. The endometriotic lesions can transform into atypical endometriosis and then into cancer. Cancers associated with endometriosis are endometrioid - and clear cell cancers.

Endometriosis is not linked to an increased risk of serous or mucinous OC. Data that the PTEN, PI3KCA and ARIDIA mutations in the atypical endometriotic lesions are identical with endometrioid and clear cell type ovarian cancer, support this hypothesis (Wiegand et al., 2010). The Ovarian Cancer Cohort Consortium analysis also supports this theory as women with endometriosis have an increased risk for the development of endometrioid (RR = 2.32, CI = 1.36–3.95) and clear cell (RR = 2.87, CI = 1.53–5.39) ovarian cancer (Wentzensen et al., 2016). Furthermore the link between retrograde menstruation and endometriosis related types of cancers is confirmed by a significant reduced incidence of endometrioid (RR = 0.60, CI = 0.41–0.88) and clear cell cancer (RR = 0.35, CI = 0.18–0.69) after tubal ligation and that there also is a significant reduced risk (43%) of clear cell type ovarian cancer after a hysterectomy (RR = 0.57, CI = 0.36–0.88) (Wentzensen et al., 2016). There could also be a protective role for a hormonal IUD. A hormonal IUD induces a high incidence of amenorrhea, subsequently a very low rate of retrograde menses and less endometriosis. Performing an operative hysteroscopy on the otherhand, provokes transtubal blood loss and could be therefore be regarded as iatrogenic retrograde menses. Theoretically this could also increase the risk of OC.

### Clinical data regarding the risk-reducing effect of tubal ligation and hysterectomy with bilateral salpingectomy

The retrograde menstruation can be blocked by tubal ligation, hysterectomy and bilateral salpingectomy with a significant reduction in the number of endometrioid and clear cell type OC as mentioned earlier (Wentzensen et al., 2016). In Belgium these cancers represent respectively 8 % and 5 % of all epithelial ovarian cancers. Additionally, the lack of decreased incidence of HGSC after a tubal ligation (RR=0.91; CU = 0.79-1.06) reflects the preservation of fimbriae in tubal ligation. Furthermore, the Ovarian Cancer Cohort Consortium analysis among 1.300.000 women showed a reduction of the overall incidence of OC by 18% (RR = 0.82, CI = 0.73-0.93). Hysterectomy however had no impact on the overall incidence of OC (RR = 0.96, CI = 0.89-1.03). A Swedish nationwide population-based study which looked at 98026 hysterectomies and OC risk showed a significant reduction in OC after hysterectomy (HR = 0.79; CI = 0.70–0.88) (Falconer et al., 2015). The difference in risk reduction by hysterectomy between the two studies is a reflection that some surgeons remove the tubes routinely during a hysterectomy and others don’t.

A bilateral salpingectomy would reduce the number of adenocarcinomas NOS (not otherwise specified) and serous carcinomas. In Belgium these cancers represent respectively 10 % and 68 % of all epithelial OC. A recent meta-analysis studied the impact of bilateral salpingectomy on OC reduction in the general population (Yoon et al., 2016a). This meta-analysis includes one cohort study and two population-based case-control studies (Lessard-Anderson et al., 2014; Madsen et al., 2015; Falconer et al., 2015). In total 3509 patients undergoing bilateral salpingectomy and 5.655.702 controls who did not undergo salpingectomy were included (Yoon et al., 2016a). Over the combined study period, 29 of the 3509 OBS patients developed OC compared with 44.006 of the 5.655.702 without salpingectomy. The results revealed that there is a 49% reduction in OC after an OBS (OR=0.51; CI = 0.35-0.75). On the contrary, while one study claimed no effect (Lessard-Anderson et al., 2014), one study showed a reduction of 42 % (OR = 0.58, CI = 0.36-0.95) (Madsen et al., 2015) and the largest study revealed a reduction of 65 % (HR = 0.35; CI = 0.17-0.73) (Falconer et al., 2015). Modelling studies showed that if the fallopian tubes were removed at the time of every hysterectomy and sterilization procedure then a potential reduction in the rate of HGSC of 40% over the next 20 years is to be expected for the operated women (Salvador et al., 2017).

### Technique of OBS in order not to compromise the blood supply

Preserving the ovarian function after an OBS is important. Premature menopause (before age 40) or early menopause (between 40 and 45 years) has been associated with an increased risk of coronary heart disease (CHD), stroke, glaucoma, cognitive impairment, dementia, Parkinson, osteoporosis, psychiatric diseases, sexual dysfunction, mood disorders and increased overall mortality (Faubion et al., 2015). Removing the ovaries during a hysterectomy before 47.5 years will reduce mortality related to ovarian and breast cancer (Parker et al., 2013). However a long-term follow-up cohort study of 30.117 women followed over 28 years showed that these benefits were neutralised by the significantly increased risks of dying from other causes: a 23% increase in CHD mortality, a 29% increase in lung cancer mortality, a 49% increase in colorectal cancer mortality and a 13% increase in all-cause mortality (Parker et al., 2013). Preserving the ovaries in women undergoing hysterectomy until the age of 55 has been associated with a mortality reduction of 8.5 % at the age of 80 (Parker et al., 2009). For this reason, hormonal therapy in women undergoing PBSO is recommended at least until the median age of the natural menopause (age 51 years).

In order to study the effect of OBS on the ovarian function it is particularly interesting to look into a group of premenopausal women undergoing ovarian stimulation as part of an IVF treatment after bilateral salpingectomy for tubal pathology. Ovarian function can be measured by looking at the ovarian follicle response, ovarian response to hyperstimulation, the ovarian reserve based on serum FSH and serum anti-Müllerian hormone (AMH) level. s-AMH is an accurate predictor of individual time-to-menopause) (Dólleman et al., 2014).

A recent meta-analysis (n = 1.482; 657 OBS and 825 no OBS) looking at the ovarian response before and after OBS in patients with IVF treatment showed there was no significant difference between the two groups regarding the peak E2 level, the total gonadotropin dose used for stimulation and the number of oocytes retrieved (Yoon et al., 2016b). Additionally, the number of pregnancies before and after OBS salpingectomy did not differ significantly.

Another meta-analysis (n = 4.828) looked at the ovarian response to hyperstimulation during IVF before and after OBS (Fan and Ma, 2016). After OBS the total dose of gonadotropin was significantly increased, the number of oocytes retrieved was significantly decreased and the FSH level was significantly increased. The conclusion of this meta-analysis was that OBS impaired ovarian response to hyperstimulation during IVF.

In a retrospective study (n = 198) the serum AMH level and FSH level were compared in women with or without an OBS during IVF treatment (Ye et al., 2015). After OBS the mean AMH level was significantly lower and the mean FSH level was significantly higher. These results suggest that OBS is associated with decreased ovarian reserve. In the study there were no significant differences in duration of gonadotropin therapy, amount of gonadotropin used, oestradiol level on the human chorionic gonadotropin injection day, thickness of the endometrium, number of oocytes retrieved, number of 2-pronuclei, viable embryos, and good quality embryos.

In a retrospective study looking at premenopausal women undergoing a hysterectomy with or without OBS there were no differences after 3 months in AMH, FSH and AFC (antral follicle count), mean ovarian diameters and peak systolic velocity (Morelli et al., 2013a). A pilot RCT (n=30; mean age 37 years) showed that OBS during a laparoscopic hysterectomy did not have an effect on the AMH levels three months after surgery (Findley et al., 2013). A larger RCT (n = 68; mean age 43 years) revealed that 3 months post-surgery the AMH levels in both groups were significantly lower than the preoperative AMH levels (Kim et al., 2018). There were however no significant differences between the both groups. In a larger multicentre RCT (n=104; age mean 44 years) there was no difference in pre-surgery and 6 months post-surgery AMH levels (Van Lieshout et al., 2018). The latter three studies showed that on the short term (3 to 6 months postoperative) at least there is no negative effect of OBS on the ovarian function after hysterectomy.

The impact on menopausal symptoms one year after a hysterectomy with or without OBS was assessed in a retrospective observational cohort study (Collins et al., 2019). The menopausal symptoms were registered by the patients in questionnaires preoperatively and at one year postoperatively. Menopausal symptoms like hot flushes, sweats or palpitations were questioned. For this analysis only data of 4906 women could be used out of the 23369 women in the register. Preoperatively there was no difference between the two groups. The overall analysis showed a significant increase in menopausal symptoms in the group who had a hysterectomy with OBS (RR = 1.29; CI = 1.04-1.60 and the adjusted RR was 1.33; CI = 1.04-1.69) (Collins et al., 2019). However, in the age-stratified adjusted analysis only women at the age of 44-69 years remain at significant risk of menopausal symptoms one year after OBS (adjusted RR = 1.53; CI = 1.06-2.20) (Collins et al., 2019).

The long term effects (3 to 5 years postoperative) of OBS on ovarian function have been described in an observational study (Venturella et al., 2017). In this study the ovarian function/age was assessed through OvAge. The latter is a statistical model that combines AMH, FSH, 3-dimensional AFC, vascular index, flow index, and vascular flow index values. The mean age at surgery and an OvAge at follow-up was respectively 45,9 and 49,3 years. The conclusion of this study was that adding OBS to a hysterectomy in the late reproductive years did not have negative effects on ovarian function 5 years after surgery.

In ‘older’ women (> 40 years) with or without an OBS there is already evidence of diminished ovarian reserve. Menopausal symptoms appear to be increased in the OBS group of 44-49 years, but this is not supported by surrogate markers like AMH, FSH, oestradiol or ultrasound (US). In these older women there appears to be no impact on the ovarian function based on these surrogate markers in the short- (3 months) and the long term (5 years).

## Safety of OBS

Several retrospective studies have looked at the safety and feasibility of OBS at the time of gynaecologic surgery. The two largest studies from Canada and the USA, evaluated respectively 43,932 and 425,180 women (McAlpine et al., 2014; Hanley et al., 2017). The studies showed no increased risk for peri- and postoperative complications, blood transfusions, length of hospital stay in days, postoperative infections or readmissions when an OBS was additionally performed during a hysterectomy (Berlit et al., 2013; Findley et al., 2013; Garcia et al., 2016; Ghezzi et al., 2009; Hanley et al., 2017; McAlpine et al., 2014; Minig et al., 2015; Morelli et al., 2013b; Song et al., 2017; Till et al., 2018; Vorwergk et al., 2014; Westberg et al., 2017). A register cohort study showed a statistically significant, though clinically irrelevant difference, in hospital stay of 2.5 hours between hysterectomy with or without BSO (Collins et al., 2019). The same group also reported a small but significant reduced amount of blood loss in the OBS group (-20 ml, CI = -40 to -0.1) (Collins et al., 2019). In addition the OBS group had significantly more minor complications after one year when adjusted for surgical route, BMI and smoking status (adjusted RR = 1.35; CI = 1.01 to 1.83) (Collins et al., 2019). However, if previous salpingitis was added then there was no longer a significant difference. There were no differences in reported severe complications overall. One study in which 540 laparoscopically assisted vaginal hysterectomies were compared with OBS (n=127) or without (n=413) revealed that there was a significant increased rate of benign adnexal pathology when the tubes were left behind (Vorwergk et al., 2014). This leads to a significant increase in surgical interventions in the non-OBS group compared to the OBS group (12.56 vs. 4.16 %; p=0.04) (Vorwergk et al., 2014).

The overwhelming majority of the studies didn’t show a difference in infection rate in the direct postoperative period. Benign tubal problems in general are rare. The annual prevalence of adnexal torsion is about 2 to 6 % (Sasaki and Miller, 2014). Rarely the fallopian tube will twist on its own, when it does it is usually enlarged (hydrosalpinx, pyosalpinx, hematosalpinx), or abnormally long, or previously ligated or associated with adhesions or paratubal cysts (Sasaki and Miller, 2014; Tjalma, 2017). The benefits of performing an OBS besides the already mentioned reduction in ovarian cancer are reductions in postoperative tubal prolapses, postoperative infections, torsions, benign and malignant tubal diseases (Basu and Ward, 2007; Ghezzi et al., 2009; Morse et al., 2002; Tjalma, 2003; Vorwergk et al., 2014). Data from the Danish cohort study showed that women who have had a hysterectomy without an OBS (n=6456) or who have had a sterilization but no OBS had a more than doubled risk of additional tubal surgery, as the OR are respectively 2.13 (CI = 1.88 to 2.42) and 2.42 (2.21 to 2.64) (Guldberg et al., 2013).

In one large study looking at the duration of surgery, performing an OBS during a hysterectomy showed significant increase in the operating time regardless which route (12 minutes; p < 0.001) (Till et al., 2018) (16 minutes; p < 0.001) (McAlpine et al., 2014). A recent RCT comparing OBS and bilateral tubal ligations at the time of caesarean delivery in women desiring permanent contraception, showed that an OBS added 15 minutes to the total operating time (p = 0.004) (Subramaniam et al., 2018). Likewise, performing an OBS instead of tubal ligation took also significantly longer (10 minutes; p < 0.001) (McAlpine et al., 2014). All other reported studies showed there was no significant difference in the operating time when an additional OBS was performed (Berlit et al., 2013; Findley et al., 2013; Garcia et al., 2016; Ghezzi et al., 2009; Minig et al., 2015; Morelli et al., 2013b; Song et al., 2017; Vorwergk et al., 2014).

In a study looking at immediate and short-term complications and surgical duration among women having laparoscopic salpingectomy (n=81) or tubal occlusion (n=68) for female sterilization there was no difference in complication rate, but the average surgical time was 6 min longer for salpingectomy compared to occlusive methods (44±13 min versus 38±15 min, respectively, p=0.018) (Westberg et al., 2017). A downside in changing from a tubal sterilization to an OBS, could mean adding additional port(s) (three ports instead of one or two) and it could also include the use of another surgical instrument with its own energy and complication rate. When OBS is considered in patients for sterilization, we need to underline the definite character of this technique as 2-5% of patients will show regret later in life, especially in younger patients (younger than 40).

In conclusion, adding an OBS to an abdominal or laparoscopic hysterectomy doesn’t increase the complications rates. It may however slightly extend the operating time. In exceptional cases, and certainly during vaginal hysterectomy, removal of the fallopian tubes is challenging. In experienced hands routine removal of free mobile fallopian tubes during abdominal or laparoscopic surgery is easily done. Removal of diseased fallopian tubes whether or not adherent to other pelvic structures could increase operation time and the complication rate. For this reason, it is important to registrate OBS and their short term and long term morbidity. Sometimes performing an OBS in these cases could mean changing the route of access (most often in case of a vaginal approach). A medical decision should be made at that time whether or not to change the route of access or to abandon the plan of OBS.

## Cost effectiveness

Several models examined the costs and benefits of OBS during hysterectomy, during caesarean delivery or surgery for permanent contraception. (Kwon et al., 2015; Dilley et al., 2017; Cadish et al., 2017; Subramaniam et al., 2019, 2018; Venkatesh et al., 2019). These studies all concluded that it is a cost-saving procedure. Prospective studies with long term follow-up are needed to measure the cost-effectiveness for the health care system.

## Consensus from other societies

OBS prevents retrograde menstruation and removes the fimbriae. OBS at the time of surgery for benign gynaecological diseases or sterilization could decrease the risk of ovarian cancer by as much as 65% (Falconer et al., 2015; Kho and Wechter, 2017; Piszczek et al., 2018). The first society to have a consensus regarding OBS was the Society of Gynaecologic Oncologists of Canada, with a consensus statement in September 2011 (SGOC, 2011; Salvador et al., 2017). Since then several societies have revealed consensus statements regarding the value of OBS during benign gynaecologic surgery (Table II). Eleven statements are in favour of OBS and five statements are ambivalent (Table II). At present, 2019, only a small minority (11%) of FIGO members have statements on opportunistic prophylactic salpingectomy (Ntoumanoglou-Schuiki et al., 2018). Consensus statements have a major impact in daily practice. After the first consensus statement publications in Canada and the USA regarding the value of OBS in the prevention of OC the uptake has increased considerably. In British Colombia (2008-2011) the uptake of hysterectomy with OBS rose from 5% (2008) to 35 % (2011) and in the USA the increase was 371 % in the period 2008-2013 (Hanley et al., 2017; McAlpine et al., 2014). In some health delivery systems like Kaiser this has led to an OBS of 73 % during a benign hysterectomy (Garcia et al., 2016).

**Table II:** — Societies with a consensus regarding OBS.

Societies in favour of OBS	
2011	Society of Gynaecologic Oncologists of Canada (SGOC, 2011)(Salvador et al., 2017).
2012	Finnish Society of Obstetrics and Gynaecology (FSOG, 2012),
2012	Dansk Selskab for Obstetrik og Gynækologi (Denmark) (FSOG, 2012)
2013,2015	Society of Gynaecologic Oncology (SGO) in the United States (SGO, 2013)(Walker et al., 2015).
2014	Royal Australian and New Zealand College of Obstetricians and Gynaecologists (RANZCOG) (updated July 2014) (RANZCOG, 2014)
2014	Royal College of Obstetricians and Gynaecologists (UK)(RCOG, 2014)
2014	Turkish Society of Gynaecologic Oncology (TSGO, 2014),
2015	American College of Obstetricians and Gynaecologists (ACOG) (Committee on Gynecologic Practice, 2015)
2015	Austrian Society of Obstetrics and Gynaecology and Austrian Society of Pathology (ASO and ASP, 2015)
2017	Japan Society of Obstetrics and Gynaecology (Fujii et al., 2017)
2018	Korean Society of Obstetrics and Gynaecology (KSOG) (Kim et al., 2018).
Societies with an ambivalent statements regarding OBS	
2015	Norsk Gynecologisk Forening (NGF, 2015)
2015	German Arbeitsgemeinschaft Gynäkologische Onkologie (AGO) (Pölcher et al., 2015)
2015	Swedish Society of Obstetrics and Gynaecology (SSOG, 2015)
2016	French College of Obstetrics and Gynaecology (Deffieux et al., 2016)
2018	National Comprehensive Cancer Network (NCCN, 2018).

In the Canadian study there was an increase in OBS for sterilization from 0.5 to 33% (McAlpine et al., 2014). By 2013 75 % of all benign hysterectomies had an OBS and 48 % of all sterilizations were OBS (Hanley et al., 2015). In Sweden the uptake of OBS at the time of hysterectomy increased since 2013. In 2012 OBS with hysterectomy in Sweden was only performed in 1.9%, in 2013 it was 8.9 % and in 2016 it increased to 37.8 % (Collins et al., 2019). The Swedish data indicate that despite the ambivalent advice of the Society (SSOG) the rates of OBS are increasing. When you compare this to Ireland, then you notice that figures haven’t changed much since the first publications regarding the role of OBS. In a questionnaire 26 % of the Irish gynaecologists indicated that they performed an OBS in 26 % of the abdominal benign hysterectomies (ABH) and in 5.4 % of the vaginal benign hysterectomies (VBH) (Kamran et al., 2013). They were willing to incorporate an OBS in 90 % of the ABH and in 66 % of the VBH as a measure to reduce cancer risk (Kamran et al., 2013).

## Conclusions and Consensus statements of the Flemish Society of OB & GYN

The paradigm shift that serous OC originate from the fallopian tube has opened the door for OBS. High grade serous OC represents more than two third of epithelial OC and is associated with advanced disease stage, high incidence of disease recurrence and poor prognosis despite aggressive surgery and advanced chemotherapy.

Current available retrospective data suggest a 49% to 65 % reduction in OC if both salpinges are removed. On the short term this procedure appears to be safe with probably a slight increase in operating time. On the long term all available evidence indicates, except for one retrospective study, that there seems to be no impact on the ovarian function/reserve when the blood supply was respected.

At present there is one ongoing randomized controlled trial looking at the outcome of OBS in asymptomatic women with an average risk on OC. This is the HOPPSA or Hysterectomy and OPPortunistic SAlpingectomy study (NCT 03045965; ClinicalTrials.gov). The HOPPSA is a register-based randomized controlled trial (R-RCT), with the objective to examine if OBS compared with no OBS, at the time of hysterectomy for a benign reason, has no increased risk of complications, has no negative side effects on ovarian function and subsequent cardiovascular disease or incidence of fractures, and implies a reduced risk of subsequent OC. Randomization and follow-up will be conducted within national registers (https://clinicaltrials.gov/ct2/show/NCT03045965).

The trial has three primary outcome measures: 1. 8 weeks post-operative surgical complication rate; 2. change in menopausal symptom score or the effects on ovarian function at 1 year; 3. the risk reduction of epithelial OC at 10 – 30 years. The study started on June 1, 2017 and the estimated time of closure is June 1, 2020. In total 4.400 participants need to be randomized and it is estimated that the study is completed for all 3 outcome parameters in 2053!

Guidelines regarding OBS by our society are urgently needed as otherwise clinicians will be caught up by (social) media and demands regarding OBS. The impact of a consensus statement by a society is high, but it protects both the patient, their families and the clinicians. Based on the principle that the benefits outweigh the risks the Flemish Society of Obstetrics and Gynaecology has formulated a statement regarding OBS (Table III).

**Table III:** — Consensus statements.

High grade serous ovarian cancer is the most common (> 65 %) form of ovarian cancer and originates most likely from the fimbriae. It is detected in stage III/IV in 78 % and has a poor 5-year survival (44%).
Endometroid (8%) and clear cell cancer (5%) are rare forms of ovarian cancer and are believed to originate in part from endometriosis. Endometriosis is caused probably by retrograde menstruation. Endometrioid and clear cell cancer are discovered in stage I in 61 % and 54 % of the cases. The detection in stage I translates in a general better survival.
Secondary prevention for women with an average risk for ovarian cancer by ultrasound or blood analysis is not indicated as it mostly increases morbidity, without reducing mortality.
Primary prevention for women with an average risk for ovarian cancer by performing opportunistic bilateral salpingectomy is likely to reduce the risk of ovarian cancer by 65 %. Due to the removal of the fimbria and the blocking of the retrograde menstruation.
Furthermore women who preserve their tubes after a hysterectomy or sterilization have a more than double risk of additional tubal surgery.
An OBS during a hysterectomy or in order to perform a sterilization is safe, has no negative impact on the ovarian function and is most likely cost effective. It may lead to prolongation of the operating time by 16 minutes.
All women undergoing abdominal surgery should be counselled about OBS when their child wish is fulfilled.
The possibility of bilateral salpingectomy as a method of sterilization should be discussed with every patient informing about sterilization and certainly an OBS should be the method of choice above the age of 40.
An OBS should be strongly considered during an abdominal (gynecological or obstetric) operation (laparoscopy (classical or robot), laparotomy or vaginal route) in women who’s child wish is fulfilled.
Patients should be explained that a salpingectomy inhibits permanently spontaneous conception.
If premature or early menopause occurs, we recommend HRT until the median age of the natural menopause (age 51 years).
In the absence of level 1 evidence, the implementation of OBS in general practice requires the prospective National registration of OBS together with the morbidity.

The outcome of the RCT will give us level one evidence regarding OBS. But it is unethical to wait for 36 years for the results of the RCT. With the evidence of OBS at the moment our Society feels that it should be introduced in daily practice together with a prospective registration. This registration project could be called PROTECT (an acronym for PROphylactic TubECTomy). This register will give us Belgian data regarding surgery-related morbidity, menopause-related quality of life, cardiovascular mortality, osteoporosis as well as ovarian cancer incidence, histopathology and cost- effectiveness of OBS. We recommend that the consensus statement is followed until there are new, evidence-based and practice changing data.
